# Assessing the adoption of biosecurity measures among extensive livestock producers: a case study in the free-range pig sector of Corsica

**DOI:** 10.1186/s12917-024-04441-w

**Published:** 2025-02-15

**Authors:** Theo Loeillot, Marie Gisclard, Bastien Trabucco, François Charrier, Ferran Jori, Nicolas Antoine-Moussiaux, Alexis Delabouglise

**Affiliations:** 1 CIRAD, UMR ASTRE, Montpellier, France; 2 UMR ASTRE, CIRAD, Université de Montpellier, INRAE, Montpellier, France; 3 INRAE, UMR AGIR, Toulouse, France; 4 UMR SELMET, Laboratoire de Recherches Sur Le Développement de L’Elevage (LRDE), INRAE, Corte, France; 5 INRAE, UMR LISIS, CNRS, Université Gustave Eiffel, Marne-La-Vallée, France; 6 University of Liège, FARAH-Fundamental and Applied Research for Animals & Health, Liège, Belgium

**Keywords:** Swine production, African Swine Fever, Pig medicine, Biosecurity, Stated preferences, Animal health economics, Free-range farming, Extensive farming

## Abstract

**Background:**

Increasingly exposed to emerging sanitary risks, extensive livestock farming systems are confronted with the imperative of incorporating biosecurity measures in their production models in order to limit the risk of introduction and diffusion of animal pathogens. Yet, ex-ante assessment methods of the likelihood of biosecurity measures implementation are poorly documented. Our study aimed at comparing alternative methods of elicitation of preferences to assess the attitude of extensive livestock farmers towards biosecurity measures. We used, as a case study, the Regional Porcine Sanitary Plan (RPSP) elaborated for the free-range pig sector of Corsica Island to meet the newly established national disease prevention requirements of France in the face of risk of African Swine Fever introduction.

**Methods:**

The RPSP imposed (1) a fencing of the breeding pigs’ area, (2) the neutering of the pigs not used for breeding, and (3) a management process of dead pigs’ carcasses found on pastures. We evaluated four attributes of the sanitary plan, including (1) proportion of the implementation cost covered by state subsidies, (2) mandatory carcass management, (3) people allowed to neuter gilts, (4) the age limit for neutering. We performed interviews of a sample of free-range pig farmers using three methods in parallel, namely (1) direct qualitative elicitation, (2) attributed-based stated choices and (3) semi-quantitative ranking of attributes.

**Results:**

Farmers’ preference for a high subsidization of the sanitary plan and for enforcing the neutering of pigs at an early age was consistent across all used methods. Participants expressed heterogeneous preferences for the two other attributes. Half of the respondents were reluctant to entrust veterinarians with neutering gilts while the other half deemed veterinarians’ intervention compulsory. Contradictory preferences were obtained on rendering carcass management mandatory depending on the elicitation method.

**Conclusion:**

Our study demonstrates the added value of using choice-based methods, where respondents weigh the individual costs and benefits associated with different options, in combination with qualitative or semi-quantitative ranking methods in which farmers express their opinions and give more consideration to their community interest. It also reveals potential issues of heterogeneities among farmers’ preferences that need to be taken into consideration in similar surveys.

**Supplementary Information:**

The online version contains supplementary material available at 10.1186/s12917-024-04441-w.

## Introduction

### Implementation of biosecurity in farms: current knowledge gap

Biosecurity refers to actions aimed at preventing the introduction of biological agents that are considered harmful for sanitary, economic or ecological reasons, in a place or in a group of animals or humans. The development of the concept of biosecurity in livestock farms has historically accompanied the intensification of agricultural systems from the middle of the twentieth century until now [1]. Indeed, the progressive confinement of animals in large numbers and the increasing use of commercial feed and other external inputs have made disease prevention actions all the more necessary as well as more feasible. Commonly recommended biosecurity actions involve, but are not limited to, a control over the entry and exit of goods, people and animals in and out of the farm area, restrictions of the contact of animals with the external environment, especially wildlife, the physical partition of animal herds according to age, species and health status, the cleaning of facilities and disposal of wastes and carcasses [2, 3]. Contrary to other more targeted health interventions like vaccination or the reporting of diseases to sanitary agencies, biosecurity entails a potentially large spectrum of actions that may complement or substitute each other, in relation to the characteristics of the farming system. Implementation of Biosecurity measures on farms is often conditioned by the type and scale of production, the herd management and the infrastructures in place. In extensive or smallholder farming systems, where animals have limited restrictions on their movements and frequently interact with wildlife [4] and domestic animals of other farms, biosecurity implementation is commonly limited.

The adoption of biosecurity measures in livestock systems has been the target of qualitative research dealing with farmers’ perception and behavior. One major contribution of these works is the contextualization of the biosecurity decisions within the economic and societal environment of farmers [5]. Biosecurity measures are indeed intricately linked with the structural constraints farmers are facing, including access to financial, educational and veterinary services [6, 7] and with the production model farmers identify themselves with [8]. Some authors have promoted the adaptation of biosecurity measures in relation with the constraints specific to each production system [9] or recommended a territorialized approach that builds on the existing local practices [10] and for the use of co-construction processes to build a common and contextualized vision of biosecurity that is shared among animal health stakeholders and is therefore most likely to be widely adopted [11]. The design of biosecurity plans in a collective way, through a collaborative process, is justified by their positive externalities: preventive actions implemented in one farm are likely to benefit neighboring herds as well by reducing the risk of pathogen transmission, while their cost is borne by the farm owner [12].

Despite the methodological progresses in the design of reliable and acceptable biosecurity plans, there is a lack of reliable tools to predict farmers’ individual decision to adopt biosecurity measures on the basis of their characteristics. Reliable decision analysis tools would enable public agencies to evaluate and select biosecurity plans on the basis of their efficacy at preventing disease transmission as well as their anticipated likelihood of being correctly implemented and maintained by farmers, despite the inherent complexity of human behavior prediction. It would also enable anticipating the extent of the adoption of biosecurity plans and the ensuing consequences on diseases’ epidemiology.

### Preference elicitation methods of biosecurity plans

Evaluating a given good requires the prior identification of the good’s attributes that supposedly will influence its adoption by stakeholders. The attributes must then be broken down into different levels that differ in a qualitative or quantitative manner. The careful identification of the attribute and their levels is a prerequisite to any preference elicitation. The attributes and their levels must be carefully determined so that the scenarios and their differences make sense to the participants and allow for the collection of information relevant to the problem being addressed. A first, direct and simple way of assessing farmers’ preference is through qualitative elicitation, by asking respondents to state their most liked and disliked attribute levels, thus constructing an ideal and worst scenario. While having the advantage of straightforwardness, this method does not weigh the relative effect of each attribute on the choice of farmers to adopt or reject a biosecurity measure. Semi-quantitative ranking has been promoted, in the field of participatory sciences, as a way of capturing the perceived relative importance of different items with a simple metric [13, 14]. Proportional piling is conventionally used for semi-quantitative ranking. It consists of the survey respondents distributing counters across the different attributes in proportion with their perceived importance for their decision making [15]. A quantitative method used in empirical economics, referred to as stated preferences, consists of confronting the respondents with fictitious choices between measures with different attribute levels. Stated preferences are based on the Lancaster’s theory of demand postulating that people’s choice among specific goods results from the additive utility attributed to each of the goods’ characteristics [16]. One such method, named attribute-based stated choices (ABSC) experiments have become a popular tool for preference elicitation when the decision involves a complex trade-off between several attributes [17]. In practice, ABSC consists in collecting choices made by a sample of participants among a set of hypothetical scenarios or goods with varying attribute levels, each hypothetical scenario being a distinct combination of the attribute levels [17]. In the field of animal health economics, ABSC were used to evaluate the preference of farmers for specific attributes of vaccination plans [18, 19], the acceptability of health surveillance systems [20, 21] and programs to reduce the use of antibiotics in livestock [22].

To the best of our knowledge, no comparative assessment of these different ways of eliciting preferences was ever attempted in the specific context of implementation of disease prevention measures on livestock farms. We aimed at filling this research gap by testing the three different methods with the same population of respondents, with the objective of evaluating the same biosecurity measures. We used the biosecurity plan introduced in the free-range pig farming sector of Corsica to mitigate the risk of transmission of African Swine Fever (ASF) as a case study. This biosecurity plan constitutes an example of an attempt at implementing radical changes in an extensive farming system with no or little pre-existing biosecurity measures, in the face of an emerging health risk.

### Context of the biosecurity plan of the Corsican free-range pig sector

Free-range farming is the dominant pig production system of Corsica. It is present throughout the Corsican territory, but mainly practiced in the mountainous areas. Since the pigs kept in this system partly feed on the forest resources during their grazing time, they are geographically tied to areas of holm oak, cork oak and chestnut groves which are common on the island [23]. The division of the value chain is very limited, most pig farmers ensuring the breeding and fattening of pigs as well as the processing and retailing of the cured meat products. Previous surveys have revealed a high heterogeneity among free-range pig farmers in their level of breeding management (grouped farrowing, fenced areas dedicated to breeding, neutering of sows not used for reproduction), use of supplementary feeding, confinement of animals (complete free range or partial fencing) and sanitation (disposal of animal waste and carcasses) [24, 25]. Two main farming organizations support the Corsican pig sector: (1) the PDO (Protected designation of origin) labelling scheme, which certifies some of the typical Corsican cured meat products , based on their geographical origin and their outdoor production system which involves the feeding of pigs on pasture and the use of the local “nustrale” pig breed [26]; (2) the GDS (“Groupement de defense sanitaire”—livestock health protection group), which implements preventive animal health policies . However, according to figures communicated in 2021, only a fraction of the free-range pig farmers were members of these organizations: approximately 150 PDO members and 300 GDS members out of a total of 651 registered farms.

In 2018, in the face of the rapid propagation of ASF in eastern and central Europe, that eventually reached the wild boar population of Belgium [27], the French government issued a ministerial decree for strengthening the biosecurity of the national pig sector [28]. ASF is a viral disease affecting wild and domestic swine, with no known risk of transmission to humans but highly contagious and lethal in both pigs and wild boar [29]. The prescribed measures of the decree chiefly aimed at preventing interactions of domestic pigs with wild boars, presumably the main channel of introduction of ASF in pig herds [30]. In practice, it implied a mandatory confinement of pigs either indoor or in outdoor enclosures delimited by an adequate fencing system. The national sanitary plan met a fierce opposition of the free-range pig sector of Corsica which deemed it incompatible with (1) the mountainous topography that makes fencing of large surfaces particularly difficult; (2) the current land tenure situation, a large fraction of the land used for pasture being public and/or exploited in common among several producers; (3) the requirement of the PDO, which prescribes the feeding of pigs on forest resources and thereby their access to large pasture areas shared between several herds [31]. Concerns were raised that the enforcement of the decree in its original form would cause free-range pig farmers to stop their activity or maintain it illegally, making disease control interventions even more difficult, as it has been described in the neighboring island of Sardinia [32].

At the time of the study, in 2021, a Regional Porcine Sanitary Plan (RPSP) was being negotiated between the state and representatives of the pig sector through a dynamic co-construction process piloted by a technical committee [11]. The RPSP was an adaptation of the national sanitary plan considering the specificities of the Corsican context. The RPSP maintained the use of free-ranging on communal pasturelands only for neutered pigs destined to slaughter and for pregnant sows, and under the condition that carcasses of dead pigs were not left on pasture for long periods. This adaptation was deemed acceptable by the different parties, considering that sexually driven interactions between domestic and wild or feral suids as well as carcasses of diseased animals would be the major causes of pathogen transmission to domestic pig populations [33]. Yet, the application of the RPSP imposed at least three costly or time-consuming adaptations to farmers: (1) the building of an appropriate fencing system for confining the breeding herd, which was inexistent or insufficient in the large majority of farms; (2) the systematic neutering of the pigs not used for breeding, meaning a generalization of oophorectomy on females; (3) the systematic collection, storing and rendering of pigs found dead on the pastures [24]. Additionally, several questions remained unanswered such as the required qualification of the persons performing the oophorectomies on farms and the age limit for neutering the pigs.

The objective of our study was to assess and compare the relevance of different methodological approaches to explore the preference of free-range pig farmers for different characteristics of the RPSP. We experimented three different methods, namely (1) qualitative elicitation, (2) ABSC and (3) semi-quantitative ranking, with a sample of free-range pig farmers.

## Material and methods

### Preliminary interviews

In order to understand the decision-making context of Corsican pig farmers, 4 exploratory semi-structured interviews were conducted with free-range pig farmers. The farmers were chosen to represent different farm categories defined in previous studies [25]. The preliminary interviews were conducted in 4 steps: (1) collection of general data on the farm in order to understand the pig farming context; (2) open-ended questions focusing on the perception and knowledge of pig health risks and biosecurity measures, their associated constraints and advantages, and reasons for implementing biosecurity measures or not; (3) open-ended questions on the future of the pig farming sector, and potential impact of biosecurity measures or of the introduction of ASF on this anticipated evolution; (4) a question on the likely response of the participant to the effective implementation of the RPSP. The interview guide is available in supplementary information 1. These preliminary interviews were used to identify the RPSP attributes of interest for the survey and to establish the survey protocol. The survey protocol was then tested by conducting 3 pilot interviews with other pig farmers and subsequently refined.

### Sampling framework

The study was carried out over the entire Corsica Island. The survey targeted a sample of 40 participants practicing free-range pig production out of a total number of free-range pig farmers estimated to be between 450 and 550 in Corsica. The selection criteria of inclusion were based on the size and stocking rate of the declared land area used for pig farming. Thus we aimed at including farmers with a stocking rate limited to 5 pigs/ha, in accordance with the PDO specifications [23] and with a minimal land area dedicated to pig farming of 12 ha [34]. Since this stocking rate was difficult to assess through simple questions, an approximation was made by considering the average number of pigs slaughtered per year and the total area used by pigs on the farm. Thus, any farm with a stocking rate lower than 1 sow per ha or less than 5 pigs slaughtered per year per ha was considered eligible for inclusion. The participants were identified through different sources, including: (1) the list of farmers collaborating with the local research center of the French National Research Institute for Agriculture, Food and the Environment (INRAE); (2) the list of farmers registered on the website of the Office of Agricultural and Rural Development of Corsica (ODARC); (3) the list of registered users of one of the main pig slaughterhouses; and (4) a respondent-driven sampling method consisting in requesting the contact of other farmers at the end of each interview.

### Protocol design

#### Evaluated RPSP attributes and attribute levels

Four attributes were selected in our study: (1) proportion of the cost covered by the state (the “subsidy” attribute), (2) dead animals management (mandatory or not mandatory), (3) people designated to perform the oophorectomies on gilts (a veterinarian not specialized in swine, a veterinarian with an expertise in swine, the farmer), (4) the age limit for neutering gilts and boars (up to 5 months or up to 9 months). Percentages of subsidies were chosen so that they would be perceived as sufficiently different by farmers, and would reflect a low (25%), medium (50%), and high (75%) coverage level of the fencing costs. The mandatory carcass management would imply several compulsory procedures: (1) the collection of dead animals twice a week, (2) storage of dead animals less than one week in a closed compartment before collection by the renderer and (3) a reporting of pig mortality. The obligation of a veterinary intervention for performing oophorectomies is justified by the (1) the use of a laparotomy and a wound stitching, (2) the use of anaesthetics that are reserved for use by physicians and veterinarians, to limit animal suffering and facilitate animal restraint, and (3) the necessity of establishing a certificate of neutering kept in the farm registry. However, it was identified as a sensitive issue during the preliminary interviews, for reasons of farmers’ claim of autonomy or mistrust of veterinarians. This attribute only concerns the neutering of females. Male castration is easier and is already common and classically performed by farmers. There remained an uncertainty on whether a veterinary intervention would be made compulsory for the neutering of females in the RPSP at the time of the study. There is currently no veterinarian specialized in pig medicine in Corsica, therefore the attribute “veterinarian specialized in pigs” is hypothetical and means that the veterinary intervention is compulsory but the farmer has the possibility to elicit the intervention of an expert in pig medicine trained and hired within the framework of the RPSP instead of a non-specialized veterinarian. The “farmer” level means the farmer is allowed to neuter the gilts himself, with no anesthesia, but with the certification of a veterinarian in charge of controlling the operation. Many pig farmers currently neuter their gilts or other farms’ gilts by themselves with no assistance. The “age” attribute for neutering was chosen because the literature [35] and preliminary interviews showed that some farmers prefer to neuter their animals late, after sexual maturity. In addition to animal welfare issues, neutering of pigs after sexual maturity leads to the possibility of having pubescent animals in the rangelands and thus a risk of sexual interaction. In order to evaluate the importance of the age limit for farmers, two levels were chosen: a mandatory castration before sexual maturity (5 months old), or a tolerance for late castrations (up to 9 months old).

#### ABSC experimental design

ABSC relies on the formulation of pairs of hypothetical scenarios, which are different combinations of attributes levels, among which participants must make a choice. The combinations of attribute levels composing the list of pairs of fictitious RPSPs were established following a D-efficient design [36]. Compared to a classical fractional factorial design the D-efficient design maximizes the number of complex questions involving a true tradeoff between attributes, i.e. a choice between pairs of alternatives having both significant advantages and disadvantages from the respondent’s perspective—for example a choice between a fictitious plan with better subsidies but higher husbandry constraints and another fictitious plan with lower subsidies but fewer constraints. In the three pilot interviews of the survey protocol, a list of questions was randomly generated following a fractional factorial design with orthogonal main effects, using the "support.Ces" package implemented in R software [37, 38]. The obtained responses were analyzed using a conditional logistic model using the “survival” R package [39]. The coefficients associated with each level of attributes as well as their standard errors were used as a prior distribution of the model coefficients to compose the optimal list used in the survey, using the "idefix" package of the R software [36]. Thirty pairs of fictional RPSP were created. Two blocks of 15 pairs were extracted, available insupplementary information 2, and each block was randomly assigned to 50% of the survey respondents.

#### Data collection process during the interviews

The interviews were conducted by the first author of the article, at the respondent’s home or any other places chosen by him. Each interview lasted between 1 and 2 h. The interview was divided into six phases. The interview guide, detailing the different phases of the interview and the questions asked to the respondent, is available in supplementary information 3. Throughout the interview, the participants' statements reflecting his opinion or attitude towards the RPSP were systematically recorded through note taking.

#### General information on the breeder and his farm

The information requested concerned the production size and land surface used, the management of the breeding and fattening stock, and the institutional context of the farm (membership in PDO and GDS organizations).

#### Introduction of the RPSP and quantification of its monetary cost

The main measures of the RPSP were first presented to the respondent. The evaluated attributes and their levels were presented. The cost of elevating the farm infrastructure and management up to the standards of the RPSP was estimated with the respondent in order for the respondent to have a clear understanding of the constraints associated with the RPSP before implementing preference elicitation. Estimates provided by the regional pig technical committee were used as price baseline values when the farmer was not able to evaluate some of the costs. The additional costs incurred per production cycle are related to breeding management: the building or upgrading of fences to delimit the breeding area where the breeding herd is confined as well as the cost of neutering of the gilts by a veterinarian. The fixed cost of fences building or upgrading was annualized according to the life span of the fences estimated by the respondent. This annualized cost of fencing was added to the annual cost of neutering the gilts.

#### Qualitative elicitation

The farmer was asked to choose the preferred level of each attribute in order to construct his fictitious “ideal” RPSP and, in contrast, the “worst” RPSP. The "ideal" and “worst” RPSPs correspond to the combination of the most favored levels and least favored attribute levels from the respondent’s perspective.

#### ABSC

The respondent was successively presented with the 15 pairs of fictitious RPSPs and asked to choose among one of the two options (A or B), considered to be the most acceptable, or the “neither” option—meaning that neither of the two RPSPs was acceptable (an example of a choice set is displayed in Table 1). The participant was informed beforehand that choosing the "neither" option meant that he or she would rather stop farming, or maintain it illegally and risk sanctions, than adopt one of the two RPSPs.

**Table 1:** Example of choice in the Attribute-Based Stated Choice experiment (ABSC) involving a pair of fictional Regional Porcine Sanitary Plans (RPSP)

Attribute	Alternative A	Alternative B	Neither
Subsidy	75%	75%	No implementation of the RPSP
Carcass management	Mandatory	Optional	
Person in charge of neutering gilts	Farmers under supervision	Non specialized veterinarian	
Age at neutering	Late tolerated (< 9 months)	Late tolerated (< 9 months)	

#### Semi-quantitative ranking

The respondent was asked to assess the relative importance of the 4 attributes in his decision making through proportional piling (PP). The respondent distributed 100 counters—in this case dry red beans – across the 4 attributes, materialized by circles on a sheet of paper, in proportion to the relative effect the respondent considered the attribute had for his decision-making [15].

#### Perception and likelihood of RPSP implementation

The respondent was asked to provide his opinion on the likelihood that he and the other pig farmers of Corsica concerned by the RPSP will actually implement the "ideal" and "worst" fictitious RPSP, should they be officially enacted.

### Data analysis

The quantitative data were analyzed using R software version 3.6.1 [38]. Data from the ABSC were analyzed with a conditional logistic model using the “survival” package [39]. A likelihood ratio test as well as a Wald test were used to assess the quality of the model. Attribute levels were evaluated based on the comparison of the odds of RPSP implementation in the presence of the “soft” level (least constraining or least costly for the farmers) compared to a baseline corresponding to the “strict” level (the most constraining or expensive for the farmer). PP results were analyzed by descriptive statistics. In order to take the direction of the individual preferences of the respondents towards the different attribute levels into account, individual results of PP for assessing the attributes’ effects on the respondents’ decisions were weighted by a −1 or + 1 coefficient (positive or negative) depending on the attribute level chosen by the respondent for his "ideal” PSPR: the “soft” levels were counted positively while the “strict” levels were counted negatively. The attribute levels “75% subsidy”, “optional carcass management”, “neutering by the farmer”, and “late neutering tolerated (< 9 months)” are considered to be the “soft” levels.

## Results

Forty free-range pig farmers participated in the survey, including 34 males and 6 females. Forty-three contacted farmers declined to participate in the survey for explicit motives of unavailability, mistrust or else. Two interviews were conducted remotely for practical reasons, one by phone and one through audio conferencing. Summary statistics on the characteristics of the participants’ farms are reported in Table 2. The production size was small and its distribution was skewed. Seventy-five percent of participants had less than 20 sows and slaughtered less than 100 pigs per year. The extensive nature of the pig production was illustrated by the low number of pigs slaughtered per unit of used land surface (1.3 pigs slaughtered per year per ha on average) despite a strong heterogeneity across farms. The breeding and fattening performances were more homogeneous, 50% of farmers slaughtering between 5.5 and 6.9 pigs per year per sow. Most participants were members of the PDO and GDS organizations. A minority of participants (28%) already practiced the neutering of females. Ninety percent of participants already had a dedicated fenced breeding area but the fencing needed to be upgraded to comply with the current requirement of the RPSP. The monetary cost of implementing the RPSP measures could be evaluated in all farms. It varied significantly across farms but was comprised between 32 and 63 EUR per slaughtered pig per year for 50% of the respondents.

**Table 2:** Characteristics of the participants’ farms: production size and performance, cost of Regional Porcine Sanitary Plan (RPSP) implementation in the farm, institutional context

Continuous measures of farm production size and performance and cost of RPSP* implementation (*n* **= 40)**	**Median**	**Minimum—maximum**
Number of sows	12	6–75
Number of slaughtered pigs per year	70	40–400
Surface used for pig grazing in hectare (ha)	100	12–500
Number of slaughtered pigs per year per hectare (/year/ha)	0.8	0.1–5.8
Number of slaughtered pigs per year per sow (/year/sow)	6	0.8–11.7
monetary cost of compliance with the RPSP* measures (EUR/slaughtered pig/year)	43	0–158
**Category of farm management and institutional context (*n*** **= 40)**	**Count**	**Proportion (%)**
Presence of a dedicated breeding area delimited by fences	36	90
Neutering of gilts	11	28
Member of the PDO** scheme	23	58
Member of the GDS***	32	80

^*^RPSP: Regional Porcine Sanitary Plan

^**^PDO: Protected designation of origin

^***^GDS: “Groupement de defense sanitaire”—livestock health protection group

The ideal fictitious RPSPs selected by participants are displayed in Table 3. Eight different “ideal” plans were chosen in total, the only consensual attribute level being the proportion coverage of the RPSP implementation cost by the state, which was systematically preferred at maximum (75%). The mandatory neutering before the age of 5 months and the mandatory carcass management were preferred by a clear majority of participants: 37 and 27 out of 40 participants (92.5% and 67.5%) respectively. The most obvious disagreement was on the person in charge of neutering the gilts: a mandatory intervention by a veterinarian and the acceptance of an intervention by the farmer under the control of a veterinarian were preferred by 21 and 19 participants (52.5% and 47.5%) respectively.

**Table 3:** Ideal fictitious Regional Porcine Sanitary Plans (RPSPs) selected by the study participants

Attributes				Number of participants
**Subsidy (%)**	**Carcass management**	**Person in charge of neutering**	**Age at neutering**	
75	Mandatory	Farmer	< 5 month	12
75	Mandatory	Veterinarian specialized in pigs	< 5 month	9
75	Mandatory	Non specialized veterinarian	< 5 month	5
75	Optional	Farmer	< 5 month	5
75	Optional	Veterinarian specialized in pigs	< 5 month	3
75	Optional	Non specialized veterinarian	< 5 month	3
75	Optional	Farmer	< 9 month	2
75	Mandatory	Non specialized veterinarian	< 9 month	1

The results of the conditional logit models applied to the ABSC data are presented in Table 4. Both the Wald test and likelihood ratio test showed that the model significantly improved the prediction of the participants' responses compared to the null model (*P* value < 0.01). However, a substantial fraction (24.3%) of the participants' responses was not correctly predicted by the model, which is attributable to a heterogeneity in participants' preferences. The coverage of the implementation cost by state subsidies had the largest effect on participants’ decision with the odds of implementation of the RPSP being 7 times higher with 75% coverage compared to 25% coverage (Odds ratio (OR) = 6.9, 95% confidence interval (CI): 4.4–10.7). Making the dead animals management optional rather than compulsory and tolerating the late neutering of pigs (before 9 months instead of 5 months) moderately increased (OR = 1.3, CI: 1–1.5) and decreased (OR = 0.7, CI: 0.6–0.9) the likelihood of RPSP implementation respectively, while the person allowed to perform the neutering on gilts was not significantly influential. Based on the result of the qualitative elicitation, we hypothesized that this absence of significant effect was due to the high heterogeneity of participants’ opinion on this specific point. Consequently, we applied the model on two sub-population: (1) participants who preferred to make the veterinarian’s intervention mandatory (*n* = 21), and (2) participants who preferred to allow farmers to perform the oophorectomy by themselves (*n* = 19). While the two subpopulations had their decision affected by the level of state subsidies, the likelihood of implementation of the RPSP was significantly increased if the farmers were allowed to perform the oophorectomy by themselves in the second subpopulation (OR = 2.8, CI: 1.8–4.5) and not significantly affected by this attribute in the first subpopulation. We also note that allowing late neutering decreased the likelihood of RPSP implementation in the second subpopulation (OR: 0.5, CI: 0.3–0.7) but not in the first one. On the contrary, not enforcing a mandatory pig carcass management by farmers increased the likelihood of RPSP implementation in the first subpopulation (OR: 1.5, CI: 1.1–1.9) but not in the second one. Finally, the second subpopulation had a higher baseline odds of not implementing the biosecurity plan than the first one (1.5 and 0.5 respectively). The model applied to the whole population of participants was used to predict the likely adoption of the RPSP by farmers depending on the level of subsidies provided by the state. All the other attributes were considered at their “strict” level, the most likely to be included in the actual RPSP (mandatory pig carcass management, oophorectomy performed by a veterinarian, neutering before 5 months). The proportion of farmers implementing the RPSP was, according to this prediction, 51.3%, 68% and 87.9% with a subsidy coverage set to 25%, 50% and 75% respectively.

**Table 4:** Results of the conditional logistic models applied to the attribute-based stated choice (ABSC) survey data

Attribute	Attribute level	All participants (*n* = 40)	Participants preferring a mandatory veterinary intervention (*n* = 21)	Participants preferring a farmer intervention (*n* = 19)
Proportion of the cost of facilities upgrading covered by subsidies	25%	Baseline	Baseline	Baseline
	50%	2**	2.9**	1.6
	75%	6.9**	12.3**	5.9**
Pig carcass management	Mandatory	Baseline	Baseline	Baseline
	Optional	1.3*	1.5**	1
Person in charge of oophorectomies	Not specialized veterinarian	Baseline	Baseline	Baseline
	Veterinarian specialized in pigs	1.1	1.4	0.8
	Farmers under the monitoring of a veterinarian	1.4	0.9	2.8**
Age at neutering	Before sexual maturity (< 5 months)	Baseline	Baseline	Baseline
	Late neutering tolerated (< 9 months)	0.7**	1	0.5**
Baseline odd of rejection of the Regional Porcine Sanitary Plan (RPSP)		0.9	0.5	1.5

The displayed odds ratios correspond to the likelihood of Regional Porcine Sanitary Plan (RPSP) adoption in the presence of the RPSP attribute level compared to the baseline level (the baseline is always the “strict” level). * and ** signs indicate statistically significant effect with p value < 0.05 and p value < 0.01 respectively

**Fig. 1:**
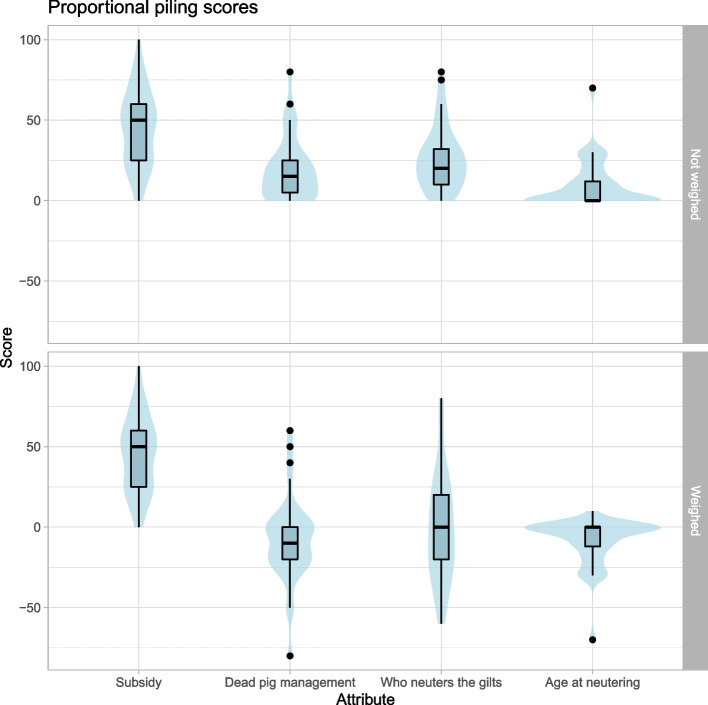
Box-and-whisker and violin plot representation of the distribution of the proportional piling scores attributed to each of the attributes of the Regional Porcine Sanitary Plan (RPSP) according to their importance for the participants’ decision

The average PP row scores (comprising only positive values) for the “subsidy”, “pig carcass management”, “person in charge of neutering” and “age at neutering” attributes were 45 (CI: 37.4–52.6), 19.4 (CI: 13.1 – 25.6), 26.5 (CI: 19.9 – 33.1) and 9.3 (CI: 4.5 – 14.1) out of 100, respectively. The average weighted scores (comprising positive and negative values depending on the direction of the preference) and 95% CI for the “subsidy”, “pig carcass management”, “person in charge of neutering” and “age at neutering” attributes were 45 (CI: 37.4–52.6), −7.6 (CI: −16.2 – 1), −0.9 (CI: −11.8 – 10.1) and −8.8 (CI: −13.6 – −3.9) out of 100 respectively. The distribution of the scores is graphically displayed in Fig. 1. The participants attributed the highest score to the level of state subsidies, which is consistent with the ABSC results. The weighted scores attributed to the person in charge of neutering were highly dispersed with an average close to 0, which is also consistent with the ABSC results and reflects the high heterogeneity in farmers’ preferences on this specific attribute. However, the participants ranked the effect of the age at neutering very low compared to the two other non-monetary attributes, which is inconsistent with the ABSC results. The participants also gave a negative score, on average, to the “pig carcass management” attribute, meaning a preference for the “strict” option of making pig carcass collection mandatory. This is consistent with the fact that the majority (27 out of 40) participants included the mandatory pig carcass management in their ideal RPSP, but it contradicts the ABSC results according to which a mandatory pig carcass management would have a negative effect on the likelihood of RPSP adoption.

The perception of the RPSP and the anticipation of its future implementation by farmers varied across participants. Eight participants (20%) had a positive perception of the RPSP because they anticipated associated benefits for the structuring of the free-range pig sector and for disease management beyond the ASF prevention, particularly in case of a highly subsidized plan. Nine participants (22.5%) were willing to implement the RPSP without legal obligation or because they considered that joining the plan would be a condition for being compensated for the losses due to ASF, should the disease be introduced in Corsica. A partial adoption of the RPSP in the population (i.e. official acceptance of the plan, but partial implementation of the measures in practice) or an increase in illegal farming (non-declaration of all or a fraction of the pigs) was anticipated by 9 farmers (22.5%), particularly in the case of a poorly subsidized plan. Five farmers anticipated that they would adopt the plan out of obligation, but that they would not fully apply it (12.5%). Seven farmers (17.5%) considered they or other farmers would not neuter all their gilts, that not all of them would be neutered by the veterinarian, or that the collection of dead pigs’ carcasses in the field would not be applied in practice.

## Discussion

In the face of the increasing frequency of emergence of new infectious animal diseases, ensuring the preparedness and resilience of livestock production while preserving the identity of the extensive and smallholder husbandry systems will become increasingly challenging. There is an urgent need to identify and assess reliable farm-level preventive measures that are effective, accepted by all stakeholders of livestock husbandry and health, and with a high likelihood of effective implementation in the extensive and smallholder systems. The case of preparedness for ASF in the free-range pig sector of Corsica provides an interesting example to test some candidate decision analysis methods for incorporation in the ex-ante evaluation of biosecurity plans and to identify the challenges associated with their usage. The study was conducted on a relatively small sample of farmers, a majority of whom where members of the GDS or the PDO, which limits the generalizability of the quantitative results to the whole free-range pig sector of Corsica. The Free-range pig sector of Corsica presents specific features, including its very extensive nature and limited structuring, that preclude any generalization of the results to other pig productions contexts [24, 25]. The study, however, allows us to make valid comparisons between the results obtained with the three elicitation methods with a view to applying them to other livestock systems.

The RPSP was elaborated in a bottom-up approach by representatives of the pig sector, animal health stakeholders and public researchers. It represents a trade-off between the imperative of elevating the level of sanitary control of the free-range pig sector while preserving its extensive model of production that valorizes the resources of the mountain forests of Corsica and provide economic opportunities to local rural actors of the island [11, 31]. Nevertheless, in the context of a poorly structured sector with substantial differences in practices and scale of production across farms, the effective implementation of the RPSP is expected to be challenging, as suggested by the study results. The question of the subsidization of the RPSP implementation by the government is expected to be critical for its adoption by farmers, a result consistent across the three used elicitation methods. This can be attributed to the relatively high estimated implementation cost, close to 50 EUR per slaughtered pig per year on average, despite important heterogeneities linked with differences in farm characteristics. For comparison, the sale price of pork is 7.5 EUR/kg on average and the finished pig carcass weight comprised between 110 and 130 kg. This result logically translates into a large difference in the modelled expected proportion of farmers adopting the RPSP in case of a 25% and 75% subsidization.

The results obtained with the three elicitation methods suggest that farmers do incorporate the non-monetary implications of biosecurity measures into their decision. The dominant preference for a mandatory neutering before sexual maturity, consistent across the three methods, may be partly driven by a concern over animal welfare. While this attribute came last in terms of importance for the farmers’ decision in the semi-quantitative ranking, its effect is significant according to the ABSC results. Consideration for animal welfare was also reported by studies conducted in other contexts [40–42]. However, in the present case, we cannot distinguish the extent of the preference for early neutering attributable to an aversion to animal suffering or to the avoidance of potential production losses resulting from late castration. The fact that a fraction of farmers already neuter their gilts, most likely without analgesia, indicates at least heterogeneous considerations for animal welfare. In addition, the opinion of the majority of farmers in favor a mandatory management of the carcasses of dead pigs could be attributed to their attentiveness towards the sanitation of the communal pasturelands.

Discrepancies were, however, observed between some attribute assessments with qualitative elicitation and semi-quantitative ranking on one side, and ABSC on the other side. These discrepancies highlight the potential contradictions that may arise in participants’ answers when questioned on actions that are desirable from a collective perspective but are costly to implement from a private standpoint. The question of the collection of pig carcasses represents a good example of this dissonance. While a large majority of the participants perceived the sanitary benefits associated with the compulsory collection and rendering of the carcasses of dead pigs by farmers, and included it in their ideal RPSP, a clear preference for an optional, rather than mandatory, pig carcass management was revealed by the ABSC results. When faced with the necessity to choose among alternative plans to implement or not in their own farm operation, farmers were probably more mindful of the individual costs, in terms of labor and organizational constraints that this mandatory carcass management would imply. Another possibility is that the minority of respondents in favor of keeping it optional were particularly sensitive to these costs when making their decision, but this explanation would contradict the outcome of the semi-quantitative ranking exercise, which gave the opportunity to participants to weight the relative importance of the attributes in their decision. It turned out that the farmers opposed to the mandatory pig carcass management did not give this attribute a higher weight than the others, resulting in a weighted average score in favor of mandatory pig carcass management, in contradiction with the ABSC results. This observation may have important policymaking implications, as an attribute viewed favorably by the stakeholders but with a limited applicability in the field may be readily incorporated in a biosecurity plan while its effective implementation by farmers would remain partial and insufficient, as suggested by the opinions expressed by the participants on the likelihood of RPSP implementation at the end of the interviews.

A strong heterogeneity was observed in the farmers’ preferences on the required qualification of the persons performing the oophorectomies, with a near equal split of the sample between two groups with opposite opinions, resulting in a non-significant preference for any of the attribute levels according to the ABSC results. This result is actually misleading, as this attribute does matter in the farmers’ individual decision, as it was ranked second on average in the semi-quantitative ranking exercise. The importance of this attribute was confirmed by applying a separate logit model on the choices performed by each of the two groups in the ABSC exercise. This heterogeneity seems to be mostly, if not completely attributable to (1) differences in the relationships of farmers with their veterinarians, the degree of trust being a critical aspect, and (2) the level of farmers’ experience in performing oophorectomies, the most experienced ones being reluctant to entrust veterinary surgeons with this task. The quality of the relationship between pig farmers and their veterinarians was partly addressed in surveys conducted in the United Kingdom or Germany, showing veterinarians are generally positively valued by farmers as a source of information and technical support for the implementation of disease control measures [40, 43, 44]. The contrasts observed in our case study can be linked with the historically low availability of veterinary services in the Corsican pig sector, compelling farmers to primarily rely on their own skills and experience for health interventions. The option of a veterinarian specialized in pig medicine did not receive a significantly higher preference than the option of a standard veterinarian. Several farmers actually preferred to involve the veterinarian they are currently working with, for reason of trust, despite his lack of expertise in pig medicine.

The study results highlight specific advantages and disadvantages associated with each elicitation method and their potential complementary role when used in combination. ABSC has the obvious advantage of being a quantitative method that enables a modelling of future decisions of farmers on the basis of the characteristics of biosecurity measures. However, its results must be considered with caution in case of limited agreement among farmers on the most acceptable characteristics – the preferred attribute levels – of the evaluated biosecurity measures. ABSC results can be misleading in this case as they tend to hide preference heterogeneities, easily overlooked by policymakers despite their implications for the implementation of biosecurity measures. While the issue of the effect of heterogeneity on the precision of the model could be addressed by ensuring a sufficiently large sample size, this is not necessarily feasible when the study population itself is small, as is the case in the Corsican pig sector. In this case, the addition of qualitative or semi-quantitative elicitations can reveal and characterize these heterogeneities that can be suitably taken into consideration in the ABSC analysis in turn. Heterogeneities can also be better characterized by analyzing associations between individual features of farmers or their farms’ enterprises and their attitude towards biosecurity, in order to make reliable inference at the population level. The second faced challenge is the potential discrepancy between the farmers’ perception of ideal collective actions on one side, evaluated from the perspective of their benefit to the whole community, and their actual choices of biosecurity plans on the other side, that appear to be more affected by the anticipation of individual costs associated with these actions. This was exemplified by the attitude of participants towards pig carcass management and the differences in the results obtained with the three elicitation methods. It points to the need of considering results of direct preference elicitations with qualitative methods with caution, as a positive perception of biosecurity actions by farmers does not necessarily mean that they would readily accept the cost of implementing them. We suggest that combining different methods of preference elicitations adequately reveal and characterize this complexity.

## Conclusion

In a context of rising vulnerability of livestock farming systems to emerging infectious diseases, reliable tools for evaluating the attitude of extensive farmers towards biosecurity measures will need to be developed and applied in the field. Preference elicitation based on stated choices is one example of a quantitative approach that could suitably fulfill this role. However, its results should be interpreted with caution in the context of heterogeneities in respondents’ preferences. We demonstrate the added value of using it in combination with qualitative and semi-quantitative elicitation methods to better characterize these heterogeneities as well as the complexity of respondents’ decisions towards measures with private costs and positive externalities.

## Supplementary Information

Supplementary Material 1. Interview guide of the preliminary interviews.

Supplementary Material 2. Choice experiment question blocks.

Supplementary Material 3. Choice experiment question blocks.

Supplementary Material 4. Respondent consent form for inclusion in the survey.

## Data Availability

The data collected in the interviews are only accessible by the researchers directly involved in the study. Any disclosure of the data to a third party should be submitted for approval to the study participants. Contact Alexis Delabouglise: alexis.delabouglise@cirad.fr.
