# Supplementary material for: Assessing the adoption of biosecurity measures among extensive livestock producers: a case study in the free-range pig sector of Corsica
Source: BMC Vet Res. 2025 Feb 15;21:69. doi: 10.1186/s12917-024-04441-w (PMC11830215; doi:10.1186/s12917-024-04441-w)
Supplement: Supplementary file 1 — Supplementary Material 1. Interview guide of the preliminary interviews. [file 12917_2024_4441_MOESM1_ESM.pdf]

Supplementary information 1

Exploratory interview guide to understand the decision-making context

Some general information about the farm :

- Number of animals and breed(s) used
- Share of pig farming in farm sales (approximate figure Ok, to give an idea of economic importance)
- Land use: types of land used (biophysical nature, resources, surface area, land tenure)
- Management of breeding and fattening (fencing, free-range grazing)
- Institutional context of the farm: PDO membership, GDS membership
- What are the benefits of these memberships (AOP, GDS, other)
- Other organizations that support the business

Feelings on the health issue:

- How do you "feel" about the health of your livestock (associated danger, economic loss, drop in performance, regulatory constraints, etc.)?
- What impact do you think swine diseases have?
- What is the impact of measures taken to combat swine diseases?
- Preventive measures implemented to reduce the risk of disease in pigs
- Herd management in the event of disease outbreaks

Feedback on biosecurity measures :

- What do you mean by biosecurity?
- What biosecurity measures are you aware of?
- (if unaware of the concept of biosecurity) What swine disease prevention measures are you aware of?
- If not mentioned, talk about fencing, castration of all butchered sows, and gestation testing for breeding sows (first the national plan, then the regional swine health plan).
- What are the constraints or advantages of adopting these measures?
- Do you have any measure in place? And why?

Feelings about the future:

- The future of the profession and the industry in Corsica
- And with the obligation to respect biosecurity measures?
- And with the arrival of ASF on the territory?

More specifically on the future of your business:

- What do you plan to do in response to the new biosecurity measures?
- Are you planning to : Bring your farm up to standard?
Stop declaring your pig farm?
Stop breeding pigs?
Other (please specify)?
- Do you prefer one of these three options? Why or why not?
- List the advantages and disadvantages associated with each option
