# Supplementary material for: Assessing the adoption of biosecurity measures among extensive livestock producers: a case study in the free-range pig sector of Corsica
Source: BMC Vet Res. 2025 Feb 15;21:69. doi: 10.1186/s12917-024-04441-w (PMC11830215; doi:10.1186/s12917-024-04441-w)
Supplement: Supplementary file 2 — Supplementary Material 2. Choice experiment question blocks. [file 12917_2024_4441_MOESM2_ESM.pdf]

Supplementary information 2

Questions from the choice experiment (block 1)

Question identifier	Attribute	Alternative A	Alternative B	Answer
1	Subsidy	25%	75%	
	Carcass management	Mandatory	Optional	
	Person in charge of neutering	Farmer	Swine veterinarian	
	Age at neutering	Late tolerated (<9 months)	Late tolerated (<9 months)	
2	Subsidy	75%	75%	
	Carcass management	Mandatory	Optional	
	Person in charge of neutering	Swine veterinarian	Non-specialized veterinarian	
	Age at neutering	Young enforced (<5 months)	Late tolerated (<9 months)	
3	Subsidy	75%	25%	
	Carcass management	Optional	Mandatory	
	Person in charge of neutering	Non-specialized veterinarian	Farmer	
	Age at neutering	Young age enforced (<5 months)	Late tolerated (<9 months)	
4	Subsidy	25%	50%	
	Carcass management	Mandatory	Optional	
	Person in charge of neutering	Farmer	Swine veterinarian	
	Age at neutering	Late tolerated (<9 months)	Must be young (<5 months)	
5	Subsidy	25%	25%	
	Carcass management	Mandatory	Optional	
	Person in charge of neutering	Non-specialized veterinarian	Non-specialized veterinarian	
	Age at neutering	Young age enforced (<5 months)	Young age enforced (<5 months)	

Question identifier	Attribute	Alternative A	Alternative B	Answer
6	Subsidy	25%	50%	
	Carcass management	Optional	Mandatory	
	Person in charge of neutering	Swine veterinarian	Non-specialized veterinarian	
	Age at neutering	Late tolerated (<9 months)	Young mandatory (<5 months)	
7	Subsidy	25%	75%	
	Carcass management	Optional	Mandatory	
	Person in charge of neutering	Farmer	Non-specialized veterinarian	
	Age at neutering	Late tolerated (<9 months)	Late tolerated (<9 months)	
8	Subsidy	50%	75%	
	Carcass management	Mandatory	Optional	
	Person in charge of neutering	Farmer	Swine veterinarian	
	Age at neutering	Late tolerated (<9 months)	Must be young (<5 months)	
9	Subsidy	75%	50%	
	Carcass management	Mandatory	Optional	
	Person in charge of neutering	Non-specialized veterinarian	Farmer	
	Age at neutering	Young mandatory (<5 months)	Late tolerated (<9 months)	
10	Subsidy	25%	50%	
	Carcass management	Optional	Mandatory	
	Person in charge of neutering	Farmer	Non-specialized veterinarian	
	Age at neutering	Young age enforced (<5 months)	Late tolerated (<9 months)	

Question identifier	Attribute	Alternative A	Alternative B	Answer
11	Subsidy	75%	75%	
	Carcass management	Mandatory	Optional	
	Person in charge of neutering	Farmer	Swine veterinarian	
	Age at neutering	Late tolerated (<9 months)	Must be young (<5 months)	
12	Subsidy	50%	25%	
	Carcass management	Optional	Mandatory	
	Person in charge of neutering	Non-specialized veterinarian	Farmer	
	Age at neutering	Late tolerated (<9 months)	Young age enforced (<5 months)	
13	Subsidy	75%	75%	
	Carcass management	Optional	Mandatory	
	Person in charge of neutering	Swine veterinarian	Farmer	
	Age at neutering	Late tolerated (<9 months)	Must be young (<5 months)	
14	Subsidy	50%	75%	
	Carcass management	Optional	Mandatory	
	Person in charge of neutering	Farmer	Swine veterinarian	
	Age at neutering	Late tolerated (<9 months)	Must be young (<5 months)	
15	Subsidy	50%	25%	
	Carcass management	Mandatory	Optional	
	Person in charge of neutering	Farmer	Swine veterinarian	
	Age at neutering	Young person mandatory (<5 months)	Late tolerated (<9 months)	

Questions from the choice experiment (block 2)

Question identifier	Attribute	Alternative A	Alternative B	Answer
1	Subsidy	75%	75%	
	Carcass management	Mandatory	Optional	
	Person in charge of neutering	Farmer	Non-specialized veterinarian	
	Age at neutering	Late tolerated (<9 months)	Late tolerated (<9 months)	
2	Subsidy	50%	75%	
	Carcass management	Mandatory	Optional	
	Person in charge of neutering	Farmer	Farmer	
	Age at neutering	Late tolerated (<9 months)	Must be young (<5 months)	
3	Subsidy	75%	50%	
	Carcass management	Optional	Mandatory	
	Person in charge of neutering	Non-specialized veterinarian	Swine veterinarian	
	Age at neutering	Young age enforced (<5 months)	Late tolerated (<9 months)	
4	Subsidy	75%	75%	
	Carcass management	Mandatory	Optional	
	Person in charge of neutering	Swine veterinarian	Farmer	
	Age at neutering	Must be young (<5 months)	Late tolerated (<9 months)	
5	Subsidy	50%	25%	
	Carcass management	Mandatory	Optional	
	Person in charge of neutering	Swine veterinarian	Farmer	
	Age at neutering	Young person mandatory (<5 months)	Late tolerated (<9 months)	

Question identifier	Attribute	Alternative A	Alternative B	Answer
6	Subsidy	25%	50%	
	Carcass management	Mandatory	Optional	
	Person in charge of neutering	Non-specialized veterinarian	Swine veterinarian	
	Age at neutering	Late tolerated (<9 months)	Young age enforced (<5 months)	
7	Subsidy	75%	50%	
	Carcass management	Optional	Mandatory	
	Person in charge of neutering	Non-specialized veterinarian	Farmer	
	Age at neutering	Late tolerated (<9 months)	Young age enforced (<5 months)	
8	Subsidy	75%	75%	
	Carcass management	Mandatory	Optional	
	Person in charge of neutering	Farmer	Swine veterinarian	
	Age at neutering	Young person mandatory (<5 months)	Late tolerated (<9 months)	
9	Subsidy	75%	75%	
	Carcass management	Mandatory	Optional	
	Person in charge of neutering	Non-specialized veterinarian	Farmer	
	Age at neutering	Late tolerated (<9 months)	Young age enforced (<5 months)	
10	Subsidy	50%	75%	
	Carcass management	Optional	Mandatory	
	Person in charge of neutering	Farmer	Non-specialized veterinarian	
	Age at neutering	Young age enforced (<5 months)	Late tolerated (<9 months)	

Question identifier	Attribute	Alternative A	Alternative B	Answer
11	Subsidy	75%	50%	
	Carcass management	Mandatory	Optional	
	Person in charge of neutering	Farmer	Swine veterinarian	
	Age at neutering	Young person mandatory (<5 months)	Late tolerated (<9 months)	
12	Subsidy	75%	75%	
	Carcass management	Optional	Mandatory	
	Person in charge of neutering	Farmer	Swine veterinarian	
	Age at neutering	Young person mandatory (<5 months)	Late tolerated (<9 months)	
13	Subsidy	25%	50%	
	Carcass management	Mandatory	Optional	
	Person in charge of neutering	Swine veterinarian	Non-specialized veterinarian	
	Age at neutering	Late tolerated (<9 months)	Young age enforced (<5 months)	
14	Subsidy	50%	75%	
	Carcass management	Optional	Mandatory	
	Person in charge of neutering	Farmer	Swine veterinarian	
	Age at neutering	Late tolerated (<9 months)	Late tolerated (<9 months)	
15	Subsidy	50%	75%	
	Carcass management	Mandatory	Optional	
	Person in charge of neutering	Swine veterinarian	Farmer	
	Age at neutering	Late tolerated (<9 months)	Late tolerated (<9 months)	
