# Supplementary material for: Assessing the adoption of biosecurity measures among extensive livestock producers: a case study in the free-range pig sector of Corsica
Source: BMC Vet Res. 2025 Feb 15;21:69. doi: 10.1186/s12917-024-04441-w (PMC11830215; doi:10.1186/s12917-024-04441-w)
Supplement: Supplementary file 3 — Supplementary Material 3. Choice experiment question blocks. [file 12917_2024_4441_MOESM3_ESM.pdf]

Supplementary information 3

Survey interview guide

1/ Explanation of the study and its objectives, with the help of the consent form.

2/ General farm data collection:

- Sale of breeding pigs
- Sales of pigs
- Number of breeding pigs
- Number of pig slaughters per year
- Used breed
- Other activities outside pig farming
- Land:
  - Types of surfaces used (biophysical nature, resources)
  - Area of the surfaces
  - Land tenure:
    - Property
    - Farming
    - Communal
  - Sharing of pastures with other breeders?
- Breeding and fattening management
  - Breeding stock:
    - Confinement
    - Partial confinement
    - Total free-ranging
  - Growing and finishing pigs:
    - Confinement
    - Partial confinement
    - Total free-ranging
- Concentrate feed: regular/irregular (4 months without feeding)?
- Concentration of births? At what period?
- Neutering of male and female pigs?
- Institutional context:
  - PDO membership
  - GDS membership
  - Farmer association membership

3/ Explain the main principles of the RPSP

- Creation of a fenced rearing area for total confinement of boars and non-pregnant breeding sows
- Neutering of pigs grown for slaughter
- Management of dead animal carcasses

4/ Collect the respondent’s general opinion on the RPSP

5/ Ask the respondent to roughly estimate the cost of implementing the health plan:

- Cost of fencing, depending on the type of fencing and the size of the planned breeding area: 13,000 EUR for a 1ha enclosure
- Cost of oophorectomy per gilt as a function of age. Veterinary intervention price: oophorectomy + travel cost of the veterinarian = 40 EUR per gilt.
- Cost of farrowing pen if necessary: 4000 EUR for 12 sows

6/ Point out that subsidy will be available, but the amount is not yet decided. In the scenarios, these subsidies will correspond to 25%, 50% or 75% of the implementation cost. This percentage of subsidy corresponds to the levels of the corresponding attribute.

7/ Show the respondent the different attribute levels

8/ For each attribute, ask the preferred level. Use it to construct the "ideal" and "worst" scenarios. Ask if they are willing to adopt the "ideal" scenario. Do the same with the "worst-case" scenario.

9/ The ABSC:

Explain that there will be 15 questions to answer, choosing between 3 alternatives. Ask if the respondent has any questions. Show pairs of randomly selected health plans one by one and ask the respondent to choose between one of the two alternatives or the "opt out" option.

10/Evaluate how important the attributes are in the respondent’s decision, using the proportional pilling method.

11/ Take the "ideal" and "worst-case" scenarios. For each of them, ask the respondent to estimate the likelihood he will implement it.

12/ Take the "ideal" and "worst-case" scenarios. For each of them, ask the respondent to estimate the likelihood that other farmers will implement it.
