# Supplementary material for: Assessing the adoption of biosecurity measures among extensive livestock producers: a case study in the free-range pig sector of Corsica
Source: BMC Vet Res. 2025 Feb 15;21:69. doi: 10.1186/s12917-024-04441-w (PMC11830215; doi:10.1186/s12917-024-04441-w)
Supplement: Supplementary file 4 — Supplementary Material 4. Respondent consent form for inclusion in the survey. [file 12917_2024_4441_MOESM4_ESM.pdf]

Informed consent form

-

Socioeconomics of African swine fever prevention measures in Corsica

-

2021

Project team members :

DELABOUGLISE, Alexis
Center International de la Recherche Agronomique pour le Développement (CIRAD)
Campus International de Baillarguet, TA-A117/E 34398 Montpellier Cedex 5, France
Email: alexis.delabouglise@cirad.fr

LOEILLOT, Théo
Intern at Centre International de la Recherche Agronomique pour le Développement (CIRAD)
Campus International de Baillarguet, TA-A117/E 34398 Montpellier Cedex 5, France
Email : theo.loeillot@supagro.fr

GISCLARD, Marie
Institut national de recherche pour l'agriculture, l'alimentation et l'environnement (INRAE)
INRAE LRDE Quartier Grossetti, 20250 Corte, France
Email : marie.gisclard@inrae.fr

Purpose of the study :

African swine fever (ASF) is a contagious disease of swine caused by a virus of the Asfaviridae family. It is particularly devastating for domestic and wild pigs, with a case-fatality rate of up to 100%. Its recent spread to Europe and East Asia is of particular concern for global food security. The presence of ASF on the neighboring island of Sardinia, its recent spread to Eastern European countries and Belgium, and the growing threat of its introduction into France have prompted the authorities to take drastic measures to bring farms up to biosecurity standards, which are due to come into force in 2021. A technical committee has proposed technical and organizational alternatives to these measures, in order to adapt them to the Corsican context of extensive free-range farming.
The aim of the study is to model the decision-making process in the adoption or non-adoption of biosecurity measures by farmers.

Topics covered :

The surveys in which you can take part will cover various topics required for the study. For example, you may be asked to provide data on your farming system and its zootechnical and economic performance, pig diseases and health management on your farm, as well as your perception of biosecurity measures and the future of your profession.

Data protection :

We guarantee that the data collected for the study will be protected.

The use of the data will be reserved for the above-mentioned study, and under no circumstances may they be delivered to private organizations or used for private purposes.

Access to survey data will be restricted to study investigators. These data will be kept confidential in a secure file.

The data analyzed will be anonymized for publication: participants' names, locations and contacts will not be mentioned.

Interrupting the interview :

The maximum duration of the interview is set at two hours, although it should last about an hour and a half. Participation in this interview is voluntary, and once you have given your consent, you can always ask for a break during the interview, or interrupt it at any time.

Survey participation :

Once you have read the above information, you may consent to your voluntary participation in the study by signing this certificate of consent:

Name of participant Date:

Signature :
